# Major incident preparedness: a curriculum and workplace necessity

**DOI:** 10.1186/s13049-020-00812-y

**Published:** 2020-12-09

**Authors:** Dominic Hampson

**Affiliations:** St Mary’s Hospital, London, Paddington W2 1NY UK

**Keywords:** Major incident preparedness, Emergency planning, Education

## Abstract

Major incidents are defined as: An event or situation with a range of serious consequences, which require special arrangements to be implemented by one, or more emergency responder agency. The ability for a healthcare system to respond effectively relies upon multiple component parts working effectively. Simulating, understanding and learning from major incidents is not widespread throughout the wider healthcare setting. However, anyone can be involved without warning. Staff working in any healthcare setting should have the knowledge and skills to respond to major incidents. It is time to include major incident response and emergency planning into the undergraduate medical curriculum. Further, it should be mandatory in all routine staff and student training. These events occur infrequently, but if managed poorly can be disastrous. This new significance placed on emergency preparedness will equip staff to face these challenges and deliver improved outcomes.

## Background

Major incidents are rare events that can cause multiple causalities to present to hospitals. The ability for a hospital to prepare and manage such events is dependent on awareness, education and rehearsal. Staff must be informed and well-practiced with a major incident plan, in order to deliver quality healthcare and reduce a potential catastrophic loss of life.

## Main text and conclusion

“Major incident declared. Go to your action station and read your action card”.

This text message or tannoy announcement may be familiar to experienced emergency medicine and intensive care consultants. However, for junior doctors, medical students and allied staff it may create confusion and panic.

Major incidents are defined as: An event or situation with a range of serious consequences, which require special arrangements to be implemented by one, or more emergency responder agency [1]. Organisations including hospitals, ambulance services and prehospital organisations respond to such incidents. Major incidents include mass casualty events such as terrorist activity, transport accidents and natural disasters. The ability for a healthcare system to respond effectively relies upon multiple component parts working efficiently. The command and control structure, communication and safety rank amongst the most important [2]. There are clinicians and resilience planners who develop and plan for these infrequent but substantial events. Organisations of all sizes, both healthcare and non- healthcare, will have bespoke plans to underpin their response [3]. However, simulating, understanding and learning from major incidents is not widespread throughout the staff who form these organisations [4]. This crucial knowledge remains limited to those who work in emergency preparedness or have experienced a major incident.

Without a basic understanding of a major incident response, how are staff supposed to respond appropriately when they find themselves in such an event? In the UK, some may believe major incidents are an urban or London phenomenon. It is unfortunately true, the majority of recent major incidents are terror related and in urban settings. However, many transport, natural disaster or CBRN incidents have occurred in rural settings and can far exceed casualty numbers of recent terror attacks. Therefore, it is not an urban healthcare problem but a common national priority.

As staff working within the National Health Service (NHS), we are all fundamentally responsible for a major incident response in some way; from direct clinical care in the emergency or intensive care departments, to medical teams creating rapid discharges to accept the wounded. One’s imagination is all that limits who could be involved. The public will expect an effective response to protect them from whichever incident lies ahead. The difficult decisions made during major incidents, will be discussed in the media and furthermore in public inquires. By neglecting this topic on an educational and safe-wide level, we could be risking poor outcomes and even public animosity towards the NHS.

It is time to include major incident response and emergency planning into the undergraduate medical curriculum. Further, it should become an integral part of workplace inductions, simulation training and postgraduate curriculum for all staff in some form. The next generation of healthcare providers should be aware of the basic principles major incident preparedness. We should all understand how a major incident plan is designed, and where we fit into the wider response. It is important to know your hospital major incident response, just like the fire drills or IT access. A major incident could be declared at any time, with a chain reaction of events and lasting ramifications for those involved. Let us be prepared and begin to embed major incident preparedness into our medical education, to improve our future responses.

## Data Availability

Not applicable.

## Abbreviations

CBRN: Chemical, Biological, Radiological and Nuclear materials
UK: United Kingdom
NHS: National Health Service
IT: Information Technology

## References

[1] Jesip.org.uk (2017). JESIP - working together, Saving Lives.

[2] NHS England (2018). Clinical guidelines for major incidents and mass casualty events.

[3] National Ambulance Resilience Unit (2019). National ambulance service command and control guide.

[4] Brennan L, Sage FJ, Simpson A (1994). Major incident planning in south East Thames region: a survey of medical staff awareness and training. Emerg Med J.

